# Effects of Different Forms of Sensorimotor Training on Postural Control and Functional Status in Patients with Chronic Low Back Pain

**DOI:** 10.3390/jpm13040634

**Published:** 2023-04-05

**Authors:** Alex Rüger, Kevin Laudner, Karl-Stefan Delank, René Schwesig, Anke Steinmetz

**Affiliations:** 1 Department of Orthopedic and Trauma Surgery, Martin-Luther-University Halle-Wittenberg, 06120 Halle, Germany; alex.rueger@uk-halle.de (A.R.); stefan.delank@uk-halle.de (K.-S.D.);; 2 Department of Ophthalmology, Martin-Luther-University Halle-Wittenberg, 06120 Halle, Germany; 3 Department of Health Sciences, University of Colorado, Colorado Springs, CO 80918, USA; klaudner@uccs.edu; 4 Department of Trauma, Reconstructive Surgery and Rehabilitation Medicine, University Medicine Greifswald, Physical and Rehabilitation Medicine, 17475 Greifswald, Germany

**Keywords:** Galileo^®^, Posturomed^®^, chronic, low back pain, sensory, motor, physiotherapy, rehabilitation

## Abstract

The aim of this study was to compare three sensorimotor training forms in patients with chronic low back pain to determine their effects on the reduction of pain-related impairment and changes in posturography. Over two weeks, during the multimodal pain therapy (MMPT) period, six sessions of sensorimotor physiotherapy or training in the Galileo^®^ or Posturomed^®^ (n = 25 per group) were performed. A significant reduction in pain-related impairment after the intervention phase was shown across all groups (time effect: *p* < 0.001; η_p_^2^ = 0.415). There was no change in postural stability (time effect: *p* = 0.666; η_p_^2^ = 0.003), but there was a significant improvement in the peripheral vestibular system (time effect: *p* = 0.014; η_p_^2^ = 0.081). An interaction effect was calculated for the forefoot-hindfoot ratio (*p* = 0.014; η_p_^2^ = 0.111). Only the Posturomed^®^ group showed an improvement in anterior-posterior weight distribution (heel load: 47% vs. 49%). These findings suggest that these forms of sensorimotor training in the context of MMPT are suitable for reducing pain-related impairment. Posturography demonstrated stimulation of a subsystem, but no improvement in postural stability.

## 1. Introduction

According to the national guideline for non-specific back pain, physical activation of the patient is important during rehabilitation, especially with the aim of maintaining activities of daily living. The inclusion of multimodal treatment concepts in the therapy of non-specific back pain should be initiated at an early stage, especially if there is a risk of chronicity or if chronicity has already occurred [1]. Interdisciplinary multimodal assessment alone seems to improve long-term outcomes for patients with chronic low back pain [2]. Sensorimotor training is often part of this multimodal approach and covers a wide range of interactions between afferent and efferent signals [3].

The aim of sensorimotor training in patients with chronic low back pain is to improve postural stabilization and function of the deep muscles (e.g., transversus abdominis muscles and multifidus muscles) and to increase intermuscular coordination with the superficial muscles (e.g., oblique abdominis muscles or latissimus dorsi muscle) [4]. In the context of three-dimensional training, Müller et al. [5] used electromyography and posturography during coordinative training in patients with non-specific back pain and showed this group had a significant improvement in intermuscular coordination of the trunk muscles and postural stability compared to patients performing passive training. As a result, a significant reduction in back pain was also observed.

Essential for muscular stabilization of the lumbar spine and for postural control is the so-called deep stabilization system, consisting of the deep musculi (Mm.) multifidi (medial tract of the autochthonous back muscles), the musculus (M.) transversus abdominis as well as the diaphragm and pelvic floor [6]. The lumbar multifidi muscles and the deep abdominal muscles, especially the transversus abdominis, are critical for the dynamic control of the individual vertebral segments and spinal stability [7,8,9,10]. As such, neuromuscular control and function of the transversus abdominis and deep multifidi muscles are often impaired in patients with back pain [11,12] and lumbar instability [13,14]. 

Various training programs targeting the deep stabilization system and thus an effector of postural control have shown good therapeutic results in patients with chronic back pain [15,16]. This is especially true for patients with back pain due to lumbar instability [17]. Tsao and Hodges [18] reported that patients with chronic low back pain who performed a single session of isolated sensorimotor training of the transversus abdominis muscle modified their electromyographic activity closer to asymptomatic patients and could cause direct improvements in the feedforward activation of this muscle even in untrained individuals and during more complex movements. They suggested that the earlier feedforward activation of the transversus abdominis in the isolated training group was potentially caused by a change in the central nervous system’s mode of preparation for posture and movement. Overall, patients with chronic low back pain have been shown to have reduced postural control compared to healthy individuals. This is especially true for more demanding balance tests [19,20]. It is assumed that critical joint positions are more likely to be adopted, which could subsequently cause injuries [3]. Researchers have concluded that sensorimotor training methods with the aim of improving postural control should be part of rehabilitative programs for chronic back pain [20] and a variety of sensorimotor training methods can be considered. These methods may include stabilizing and coordinating exercises within the sensorimotor system, as well as other therapeutic instruments such as Posturomed^®^ and Galileo^®^. 

Posturomed^®^ is used to keep the freely swinging surface as still as possible during the exercises and to make as few compensating movements as possible [21]. The physical principle of action is that the neuromuscular system must adapt to the resulting oscillation frequencies (i.e., intercept its own movement impulses (feedforward mechanism)). The aim is to increase the performance of the postural system and consequently improve posture and balance regulation. During training with the Galileo^®^ platform, the vibrating platform (10 Hz) causes reflex-controlled muscle contractions via the stretch reflex of the muscle spindles and their afferent receptors. These reflex contractions then help in postural stabilization. The Meissner corpuscles are responsible for the perception and processing of low-frequency vibrations, and the Vater-Pacini corpuscles are responsible for high-frequency vibrations. At the same time, vibrational stimuli also have inhibitory effects on the neuromuscular system [22]. A significant improvement in proprioceptive properties in the lumbar region [23] as well as a reduction in pain intensity and an increase in postural stability in patients with chronic back pain [24,25,26] have been demonstrated. 

The aim of this study was to compare the effects of sensorimotor training on postural control and pain-related impairment in the context of inpatient multimodal pain therapy (MMPT) among patients with chronic non-specific low back pain, using training on the Galileo^®^ or the Posturomed^®^ in comparison with conventional physiotherapy (PT) focusing on sensorimotor training.

## 2. Materials and Methods

### 2.1. Participants

Notably, 75 participants were included in this prospective, randomized, confirmatory intervention study and randomly assigned to 1 of the 3 training groups. During a two-week inpatient MMPT stay, the participants were each given six sessions of sensorimotor training in Galileo^®^, Posturomed^®^, or traditional sensorimotor physiotherapy training. The participants were examined pre-intervention and postintervention using the Oswestry Low Back Pain Questionnaire and posturography (Figure S1). The initial orthopedic examinations took place between July 2015 and January 2017 in an orthopedic ward of the university hospital in Halle (Saale). To be included, participants had to be 18 years old or older and diagnosed with chronic low back pain present for greater than 12 weeks. Exclusion criteria included a history of trauma, infection, tumor, or operation of the spine as well as pregnancy or breastfeeding. Current imaging was available for all patients, so low back pain requiring surgical intervention, e.g., radiculopathies or relevant instabilities, was also excluded. The MMPT included an individual combination of physiotherapy, manual medicine, interventional pain therapy, medical training therapy, relaxation techniques, oral pain medication, psychoeducation, and psychotherapy with at least 30 therapy units. The study was conducted according to the Declaration of Helsinki. Ethical clearance was granted by the Institutional Ethics Committee (reference number: 2015-84), and written informed consent was obtained from all participants prior to any data collection.

### 2.2. Oswestry Low Back Pain Questionnaire

The Oswestry *Low Back Pain Questionnaire*, also in the 2006 German translation, is an established instrument to assess the pain and functional status of patients with chronic low back pain [27,28]. Participants’ pain-related limitations in various everyday activities, such as personal hygiene, sitting, sleeping, or traveling, were surveyed using the Oswestry Disability Index (ODI). This index indicates that patients with mild limitations in activities of daily living have a degree of limitation from 0 to 20%, moderate from 21 to 40%, severe from 41 to 60%, disabling from 61 to 80%, and bedridden from 81 to 100%.

### 2.3. Posturography

Posturography is an established method for identifying training effects in the area of balance regulation in various settings, including rehabilitation [29]. The Interactive Balance System (IBS) (neurodata GmbH, Wien, Austria) was used to measure postural stability and regulation. This assessment differentiated vertical forces in the forefoot and hindfoot area on two force measurement platforms (each with a forefoot and hindfoot plate) at an anteriorly open angle of 30° using strain gauges. As a stimulus response, body sway was determined by the force measurement platforms using different parameters (Table S1) [30,31]. Compared to an asymptomatic population, back pain patients conspicuously present with higher values in the stability indicator (ST), which suggests greater instability and a comparatively insufficient peripheral vestibular system (frequency band F2–4) [32].

### 2.4. Interventions

#### 2.4.1. Sensorimotor Physiotherapy Training

For PT, a total of six training sessions were completed in the PT group (n = 25). This group performed all six sessions under physiotherapeutic supervision by an experienced physiotherapist involved in each case. The total training time per session was 15 min and included a series of exercises using both stable and unstable surfaces (Table S2). 

#### 2.4.2. Gallileo^®^ Training

A Galileo^®^ Med M vibration platform (Novotec Medical GmbH, Pforzheim, Germany) (Figure S2) was used for training the Galileo^®^ group (n = 25). This therapy platform uses a side-alternating movement pattern such as a seesaw with variable amplitude and frequency. For this training, a tilting movement of the pelvis generates a movement pattern, similar to a human gait, but much more frequent. The body reacts in a compensatory manner with rhythmic muscle contractions, which are reflex controlled from a frequency of approximately 10 Hz. This activates the muscles in the legs, abdomen, and back throughout the torso. The frequency of the vibration plate is continuously adjustable between 5 and 30 Hz, as is the amplitude of ±4.5 mm. Low frequencies can be used for mobilization, medium for muscle function and coordination, and high to increase muscle performance and endurance. 

A total of six training sessions were carried out by the Galileo^®^ group under physiotherapeutic supervision by one of two experienced physiotherapists. The total training time per session was 15 min and included a warm-up/acclimatization task, followed by 6 exercises (Table S3). 

#### 2.4.3. Posturomed^®^ Training 

The Bioswing Posturomed^®^ 202 platform (Haider Bioswing GmbH, Pullenreuth, Germany) (Figure S3) was used for training the Posturomed^®^ group (n = 25). This is a sensorimotor therapy and training device with a 60 × 60 cm damped oscillating unstable surface. The surface, which is suspended from eight steel cables, enables progressively damped (i.e., as the deflection of the therapy surface increases, the damping/resistance to deflection increases) evasive movements. Different oscillation amplitudes (max. surface deflection mediolateral 25 mm (locked)/50 mm (unlocked); max. surface deflection anterior-posterior 25 mm (locked)/50 mm (unlocked)) can be achieved by unlocking additional ropes. Maximum surface deflection in the anterior-posterior directions (40 mm/80 mm) and oscillation frequencies (1.0 to 4.2 Hz) are enabled. This allowed for an ideal severity adjustment of the exercises. 

The exercises performed by the Posturomed^®^ group consisted of six total training sessions, which were performed under physiotherapeutic guidance. The total time per training session was 15 min and consisted of a consecutive sequence of nine sensorimotor exercises on the Posturomed^®^ platform (Table S4). Two 30 s exercise periods and 30 s rest periods were planned for each level. 

If a performance error occurred in any of the groups during a specific exercise, then an additional repetition(s) was performed until the exercise was performed safely and correctly. The progress in the exercises was decided individually based on the physiotherapists’ assessment of the participant’s abilities and correct execution of the elements.

### 2.5. Statistics

Data were analyzed using the statistical program SPSS 28.0 for Windows (SPSS, Chicago, IL, USA). Analyses of variance were performed to compare mean values or detect any relationships. 

For the sample size calculation (power analysis), this study was based on the results of a comparable study design [24]. In this previous study, 2 groups with 25 participants per group suffering from chronic, non-specific back pain were compared with regard to vibration training. An effect size for the ODI of d = 0.72 was obtained in this work. The goal was to achieve a similar medium-to-high effect for each group and variable. In this regard, according to Bortz [33], with an effect size of 0.7 (α = 0.05; 1 − β = 0.8), 25 participants per group were required to obtain a relevant change. 

The critical level of significance was adjusted using the Bonferroni correction. For the Bonferroni correction, a significance level (*p*) of 0.05 was divided by the number of total tests [10]. As such, differences between means were considered significant, if *p* values were <0.005 or partial eta squared (η_p_^2^) values were greater than 0.15 [34].

## 3. Results

### 3.1. Participants

A total of 75 participants (women: n = 48 (64%)) equally divided into 3 groups (n = 25) were included in this study. Anthropometric parameters such as age, height, weight, and body mass index (BMI) did not show any significant differences between groups (Table 1).

### 3.2. Oswestry Disability Index (ODI)

**Figure 1:**
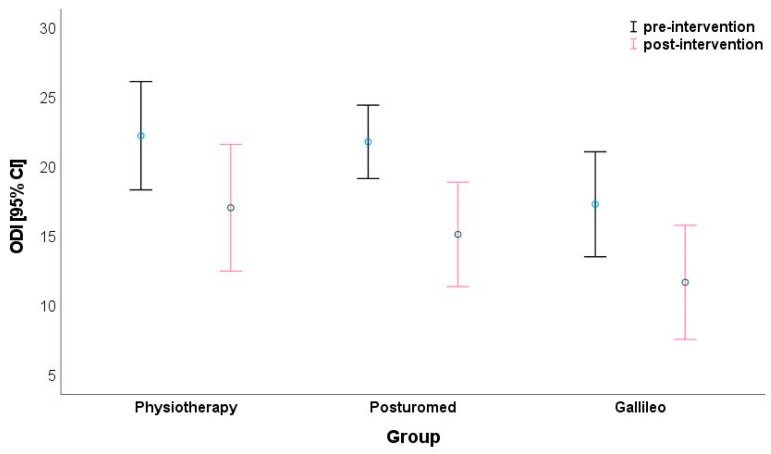
Comparison of the Oswestry Disability Index (ODI) depending on group and time (time effect: *p* < 0.001; η_p_^2^ = 0.470).

The evaluation of ODI showed a significant reduction in pain-related limitations for all groups following the two-week inpatient stay. Thus, the mean total ODI scores of the PT group decreased from 22.3 ± 2.32 to 17.1 ± 8.57 (d = 0.95). In the Galileo^®^ group, these scores decreased from 17.3 ± 7.10 to 11.7 ± 7.72 (d = 0.76) and in the Posturomed^®^ group, from 21.8 ± 5.81 to 15.1 ± 8.27 (d = 0.95) (Figure 1). In the ODI, this translates to a mean reduction in limitations from 45% to 34% for the PT group, from 35% to 23% for the Galileo^®^ group, and from 44% to 30% in the Posturomed^®^ group. From this, an improvement in the level from severe to moderate pain-related limitation can be shown for the PT and Posturomed^®^ groups. The Galileo^®^ group moves from the upper to the lower limit of moderate limitation. Large effect sizes for the reduction of pain-related limitations were found for all three groups, with those of the PT and Posturomed^®^ groups exceeding those of the Galileo^®^ group. Analysis of variance showed a strong time effect (*p* < 0.001; η_p_^2^ = 0.470) for the reduction of pain-related limitations from pre- to postintervention. There were no cross-sectional baseline differences between the groups (*p* = 0.086; η_p_^2^ = 0.094). However, there was also no significance demonstrated for the interaction effect (i.e., a difference in time trend between groups (time × group)) (*p* = 0.763; η_p_^2^ = 0.011) (Table 2). However, for the individual pre-/postintervention developments, the largest effect sizes were found in the range of a medium or large effect according to Cohen’s d (medium effect d = 0.5–0.8, large effect d > 0.8), especially in areas that the participants could easily experience during the inpatient stay. Thus, medium to large effect sizes (d = 0.65–1.61; *p* < 0.001; η_p_^2^ = 0.415) were observed for pain intensity across all groups over time and likewise for impairment during standing (d = 0.64–0.75; *p* < 0.001; η_p_^2^ = 0.338). However, again, no significant differences were observed in the cross-section of each group or between groups over time for pain intensity (*p* = 0.937; η_p_^2^ = 0.002 (group); *p* = 0.053; η_p_^2^ = 0.078 (time × group)) or impairment on standing (*p* = 0.287; η_p_^2^ = 0.034 (group); *p* = 1.000; η_p_^2^ = 0.000 (time × group); Table 2).

### 3.3. Posturography

**Figure 2:**
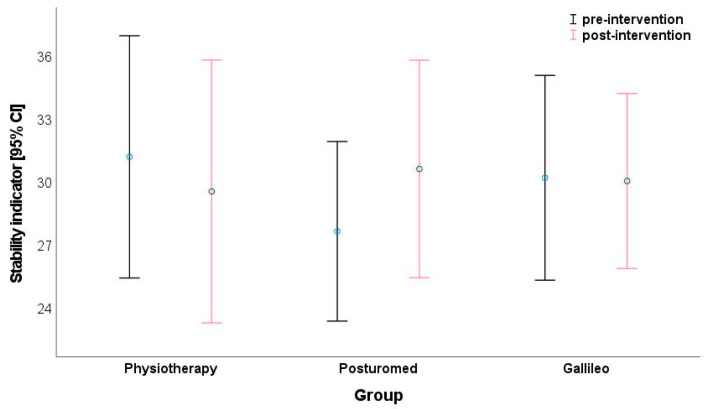
Comparison of stability indicator depending on group and time (time effect: *p* = 0.666; η_p_^2^ = 0.003).

For posturography, no significant improvements in postural control were calculated in any of the three groups over the measurement period. The stability indicator as a parameter of postural stability (the larger the value, the higher the instability) showed no significant change in the PT group from 31.2 to 29.6 (d = 0.11), in the Galileo^®^ group from 30.2 to 30.1 (d = −0.01) and in the Posturomed^®^ group from 27.7 to 30.6 (d = −0.26) (Figure 2). No significant changes were found for the stability indicator, group effect (*p* = 0.927; η_p_^2^ = 0.002), time effect (*p* = 0.666; η_p_^2^ = 0.003), and interaction effect (*p* = 0.109; η_p_^2^ = 0.060; Table 3).

In the respective frequency ranges one to eight, there was a weak significant effect for time, but only for frequency band F2–4, which maps the peripheral vestibular system (*p* = 0.014; η_p_^2^ = 0.081). There was no effect for this frequency band by group (*p* = 0.723; η_p_^2^ = 0.009) or for group x time (*p* = 0.767; η_p_^2^ = 0.007). Another significant effect was found for the interaction over time for heel loading (*p* = 0.014; η_p_^2^ = 0.111). Here, the participants in the PT group moved from 48% to 46% (d = 0.32) towards an increased forefoot load, while those in the Posturomed^®^ group moved from 47% to 49% (d = −0.30) towards an increased rearfoot load. The participants in the Galileo^®^ group showed little change in terms of heel loading from 47.4% to 47.2% (d = 0.02; Table 3).

## 4. Discussion

The reduction of the ODI scores within this study across all groups shows the effectiveness of the different sensorimotor training forms and, in connection with their effectiveness, that of the MMPT, for a reduction in pain-related limitations in patients with lower back pain. Under comparable baseline values, there was no significant difference in the reduction of pain-related limitations between the individual groups. These results suggest that, on the one hand, sensorimotor training using physiotherapeutic exercises is equivalent to training on the Galileo^®^/Posturomed^®^ platforms. On the other hand, these devices can also be a supplement for sensorimotor training, as they have similar effects on the reduction of pain-related limitations in chronic low back pain. It should also be noted that although the individual parameters of the ODI are also shown in Table 2, these have no relevance in the evaluation, since the ODI is evaluated as an overall questionnaire and no evaluation of the individual questions took place independently of the overall result.

Several studies have already investigated the effects of sensorimotor training on the subjective, pain-related limitation of participants measured by the ODI. Significant reductions in pain-related limitations are shown in many studies [24,25,35,36]. Yang and Seo [25] compared a group using only sensorimotor physiotherapy training with a group combined with vibration training. As in our study, there was a significant reduction in the ODI values for both groups, but no significance for an effect in the development over time of each other could be demonstrated. 

However, other studies did not demonstrate any positive effects of sensorimotor training in the reduction of pain-related impairment [37,38]. This is contrary to the results obtained in our study. For example, McCaskey et al. [37] did not find a significant reduction in ODI scores for either the intervention or control group during a four-week training with a total of nine 45-min interventions. In comparison, this constellation resembles the exercise design of the interventions performed in this study with respect to both the sequence with supplemental physical therapy and the intervention duration on the Posturomed^®^, as well as the low total number of exercises (single digits). However, the baseline ODI represents a significant difference with similar anthropometric conditions in the study populations. In McCaskey et al. [37] during the pre-intervention, there was an ODI percentage limitation of 20% for participants in the intervention group and 18% for those in the control group. In contrast, baseline values in our study were 45% for the physiotherapy group, 44% for the Posturomed^®^ group, and 35% for the Galileo^®^ group. If the results of del Pozo-Cruz et al. [24] are also considered, where the baseline values were 27% (intervention) and 29% (control group), it is conceivable that the comparatively lower baseline values did not allow for a significant effect. This is also suggested by Wegener et al. [38] who reported no significant reduction in ODI scores, even over a longer intervention period of 18 weeks with two training sessions per week. Their baseline ODI values were 21% in the physiotherapy group (in terms of sensorimotor training) and 18% in the vibration training group.

The results of this study showed that there were no significant positive effects on postural control as detected by posturography, for sensorimotor physiotherapeutic training, Galileo^®^ training, or Posturomed^®^ training. This is contrary to the majority of studies that demonstrated significant postural improvements following sensorimotor physiotherapeutic training and sensorimotor vibration training [5,24,26,38,39,40,41,42,43,44]. However, it is difficult to relate the results of these studies to the current study. This is because the measurements of postural control across the various studies were conducted using different measurement systems. Only two studies used the IBS [5,39] which was used in the current investigation. In the majority of subsequent studies, the ability to balance on a Posturomed^®^ or gait belt was measured using electromyographic recordings as a representation of postural control [37,40,41,42]. Other studies have used the Biodex Balancesystem^®^ [24], the Bretz Stabilometer^®^ [44], the Leonardo Mechanograph^®^ [36], or the MFT S3 Check^®^ [38] for this same purpose. Although all of these studies did mention that their respective balance system was a valid assessment tool of the postural system. Unfortunately, these differences in instrumentation make comparing results across studies difficult.

Due to similarities in methodology, the results of this study can be directly compared with the study conducted by Schwesig et al. [39]. These authors investigated the effects of three months of sensorimotor training (15 min per week on an unstable mat) among osteoporosis patients. This group was then compared to an asymptomatic control group, which individually completed daily sensorimotor training at home according to the authors’ instructions. This study reported that the postural performance, measured by the stability indicator, significantly improved by 80% of the participants in the osteoporosis group and 75% in the control group [39]. This suggests that an individual continuation of sensorimotor training at home among patients with low back pain may also provide positive results. The importance of this assumption can be concluded from the fact that Schwesig et al. [39] again showed a significant deterioration in postural performance for the three-month and two-year follow-up without sensorimotor training intervention, which was even greater in the osteoporosis group compared to the control group. 

The results of this study showed that all three groups had a significant improvement in the performance of the peripheral vestibular system, without any effect between them over time. This speaks to the comparability of the three sensorimotor training methods, as already established in the development of the scores of the ODI. Lauenroth et al. [32] previously reported that this postural subsystem shows an insufficiency in back pain patients compared to the asymptomatic population. Unfortunately, there are currently no comparative data for sensorimotor training in chronic back pain patients. Nevertheless, this development shows that the postural system was at least stimulated with the completed sensorimotor trainings, even if not yet with a direct influence on the stability indicator. Thus, it can also be assumed from this result that a longer training duration and a combination of different or very varied sensorimotor training methods, in order to challenge the postural system, would be more likely to bring about an improvement in postural control as measured by the IBS. To preserve an achieved improvement in postural control, a permanent execution of the exercises seems to be indispensable. However, future research is necessary to determine which training methods or combinations with the corresponding training intensities are necessary for improvement. 

A similar conclusion was reached by Jafarzadeh et al. [45] who compared sensorimotor training against the same training with transcranial direct current stimulation of the motor cortex. When compared to the current study, Jafarzadeh et al. [45]’s study had a similar duration and number of training sessions, but their low back pain patients were much younger. Similarly to the current study, Jafarzadeh et al. only found an improvement in postural control among the group that received transcranial direct current stimulation in addition to the sensorimotor training [45]. Because of the differences in patient ages between these studies, it is important for future research to determine whether the results presented by Jafarzadeh et al. can be reproduced in older patients who are more likely to suffer from chronic back pain.

### Limitations

One unique feature of this current study was the inpatient setting. Since all patients were participating in an inpatient MMPT, there was potentially a higher degree of limitations in activities of daily living compared to patients in an outpatient setting. This is comparable to other sensorimotor training studies which used outpatient chronic pain patients [24,37] or persons without a history of back pain [42,43].

During the recruitment of study participants, there was no specific questioning about diseases that may have had an influence on the postural system and its subsystems [3], such as Parkinson’s or cerebellar disorders [31]. Another factor that influences postural control is the degree of physical activity of the individual participants [3], which was not included in the study questionnaire.

Double blinding was not possible due to the initial fixed randomization list, since both the investigators/treatment providers and the participants could recognize the corresponding group assignment via their intervention. The participants were assigned to the next free place in the randomization list one after the other, depending on their inclusion time. If a loss-to-follow-up occurred, the vacant place in the randomization list was assigned to the next available participant included in the study, without categorizing the cause of the loss-to-follow-up or the number of dropouts per group or in total.

Three groups were compared in this study, each with an intervention of sensorimotor training using Galileo^®^, Posturomed^®^, or a physiotherapeutic training program. Consequently, there was no untrained control group, which could have led to a bias of results due to a more intensive or additive therapy of additional sensorimotor training of the intervention groups. Thus, it is likely that the observed effects with respect to the reduction of pain-related impairment by means of the ODI over the two-week intervention phase are, at least in part, effects co-induced by the MMPT. This point is particularly unremarkable since this is precisely one of the goals of MMPT. 

## 5. Conclusions

Sensorimotor training in the context of multimodal pain therapy is suitable for reducing pain-related impairment. Posturography shows stimulation of a subsystem, but no improvement in postural stability. Sensorimotor training on the Galileo^®^ or Posturomed^®^ platforms is equivalent to a physiotherapeutic sensorimotor exercise program. Therefore, these devices can be a supplement for sensorimotor training, as they have similar effects on the reduction of pain-related limitations in chronic back pain.

## Figures and Tables

**Table 1:** Demographic and anthropometric data of the sample (n = 75). Values are given as mean ± standard deviation.

Group	Body Height [m]	Age [Years]	Body Weight [kg]	BMI [kg/m]^2^
PT	1.68 ± 0.07	59.9 ± 10.7	81.7 ± 15.6	29.1 ± 5.34
Galileo^®^	1.70 ± 0.10	58.3 ± 11.6	79.6 ± 19.2	27.6 ± 5.42
Posturomed^®^	1.72 ± 0.12	62.0 ± 11.8	87.2 ± 15.9	29.5 ± 4.76
*p*	0.343	0.529	0.277	0.385
η_p_^2^	0.029	0.018	0.035	0.026

PT: conventional physiotherapy; *p*: significance level.

**Table 2:** Descriptive group comparison (mean ± SD) using the Oswestry Disability Index.

Oswestry Disability Index															
Parameter	Physiotherapy Group			Galileo^®^ Group			Posturomed^®^ Group			Variance Analyses (ALM)					
	Pre-Score	Post-Score	d	Pre-Score	Post-Score	d	Pre-Score	Post-Score	d	Group		Time		Time × Group	
										*p*	η_p_^2^	*p*	η_p_^2^	*p*	η_p_^2^
Pain intensity	2.44 ± 0.65	1.60 ± 0.82	**1.14**	2.36 ± 0.76	1.80 ± 0.96	**0.65**	2.72 ± 0.68	1.40 ± 0.96	**1.61**	0.937	0.002	**<0.001**	**0.415**	0.053	0.078
Body care	0.92 ± 0.95	0.44 ± 0.65	**0.60**	0.72 ± 0.74	0.52 ± 0.65	0.29	0.96 ± 0.98	0.44 ± 0.65	**0.63**	0.910	0.003	**<0.001**	**0.212**	0.298	0.033
Gait	1.28 ± 1.17	0.88 ± 0.93	0.38	1.12 ± 0.83	0.76 ± 0.88	0.42	1.40 ± 1.12	1.08 ± 0.99	0.32	0.492	0.020	**0.001**	**0.141**	0.953	0.001
Sedentary lifestyle	2.28 ± 1.06	2.04 ± 1.17	0.21	2.04 ± 0.79	1.44 ± 1.04	**0.65**	2.48 ± 0.92	1.76 ± 0.97	**0.76**	0.161	0.050	**<0.001**	**0.199**	0.259	0.037
stand	2.80 ± 0.87	2.00 ± 1.26	**0.75**	2.40 ± 1.04	1.60 ± 1.29	**0.68**	2.80 ± 1.12	2.00 ± 1.38	**0.64**	0.287	0.034	**<0.001**	**0.338**	1.000	0.000
sleep	1.68 ± 1.03	1.20 ± 0.91	0.49	1.72 ± 1.02	1.40 ± 1.04	0.31	1.80 ± 0.91	1.04 ± 0.68	**0.95**	0.798	0.006	**<0.001**	**0.219**	0.297	0.033
Sexuality	2.06 ± 2.08	1.69 ± 1.92	0.19	1.56 ± 1.50	0.88 ± 1.46	0.46	2.19 ± 1.63	1.33 ± 1.62	**0.53**	0.465	0.030	**<0.001**	**0.245**	0.451	0.031
Social life	2.36 ± 1.25	1.64 ± 1.47	0.44	2.08 ± 1.22	1.20 ± 1.32	**0.69**	2.16 ± 1.14	1.24 ± 1.09	**0.82**	0.456	0.022	**<0.001**	**0.310**	0.843	0.005
Travel	2.52 ± 1.33	1.40 ± 1.29	**0.85**	1.92 ± 1.58	1.08 ± 1.12	**0.62**	2.48 ± 1.39	1.76 ± 1.62	0.48	0.165	0.049	**<0.001**	**0.276**	0.619	0.013
Lifting	2.76 ± 1.20	2.28 ± 1.49	0.36	1.96 ± 1.24	1.72 ± 1.40	0.18	2.80 ± 1.16	2.20 ± 1.53	0.45	0.084	0.066	**0.002**	**0.128**	0.545	0.017
Sum	22.3 ± 2.32	17.1 ± 8.57	0.95	17.3 ± 7.10	11.7 ± 7.72	0.76	21.8 ± 5.81	15.1 ± 8.27	0.95	0.086	0.094	**<0.001**	**0.470**	0.763	0.011

Analysis of variance (relevant differences marked in bold), calculation of effect size (η_p_^2^). Scores of sensorimotor interventions (physiotherapy (PT), Galileo^®^, and Posturomed^®^). Values are given as mean ± standard deviation for the single categories and the summary score. Variance analysis (group, time, interaction effects) for the two weeks of multimodal pain therapy. ALM: General linear model (*p*, η_p_^2^); effect size (d).

**Table 3:** Descriptive group comparison (mean ± SD) using posturography.

Posturography															
Parameter	Physiotherapy Group			Galileo^®^ Group			Posturomed^®^ Group			Variance Analyses (ALM)					
	Pre	Post	d	Pre	Post	d	Pre	Post	d	Group		Time		Time × Group	
										*p*	η_p_^2^	*p*	η_p_^2^	*p*	η_p_^2^
F 1	19.4 ± 9.07	18.0 ± 6.59	0.17	17.7 ± 5.59	18.1 ± 5.49	−0.08	18.4 ± 6.61	23.6 ± 25.6	−0.33	0.467	0.021	0.426	0.009	0.315	0.032
F 2–4	11.9 ± 3.99	10.3 ± 3.41	0.42	11.6 ± 3.64	10.8 ± 2.78	0.26	11.0 ± 5.15	10.1 ± 2.11	0.25	0.723	0.009	**0.014**	**0.081**	0.767	0.007
F 5–6	5.63 ± 2.37	5.08 ± 2.30	0.24	5.29 ± 1.89	5.38 ± 1.85	−0.05	4.80 ± 1.69	5.17 ± 1.92	−0.20	0.748	0.008	0.844	0.001	0.050	0.080
F 7–8	1.06 ± 0.55	1.04 ± 0.55	0.04	0.96 ± 0.44	1.02 ± 0.37	−0.15	0.92 ± 0.37	1.07 ± 0.53	−0.33	0.884	0.003	0.59	0.049	0.103	0.061
ST	31.2 ± 14.0	29.6 ± 15.2	0.11	30.2 ± 11.8	30.1 ± 10.1	−0.01	27.7 ± 10.4	30.6 ± 12.6	−0.26	0.927	0.002	0.666	0.003	0.109	0.060
WDI	6.27 ± 2.52	5.64 ± 2.34	0.26	5.67 ± 2.27	5.32 ± 2.04	0.16	5.06 ± 1.41	5.46 ± 1.75	−0.25	0.424	0.024	0.341	0.013	0.103	0.061
Synch.	672 ± 155	636 ± 145	0.24	635 ± 128	634 ± 132	0.01	670 ± 139	644 ± 120	0.20	0.781	0.007	0.117	0.034	0.551	0.016
Left	49.6 ± 6.76	50.1 ± 4.19	−0.09	51.5 ± 4.74	50.9 ± 3.88	0.13	50.5 ± 4.67	49.0 ± 5.48	0.30	0.468	0.021	0.226	0.020	0.174	0.047
Heel	47.9 ± 7.51	45.5 ± 7.20	0.32	47.4 ± 7.73	47.2 ± 8.22	0.02	46.7 ± 5.92	48.7 ± 7.35	−0.30	0.886	0.003	0.781	0.001	**0.014**	**0.111**

Analysis of variance (relevant differences marked in bold) and calculation of effect size (η_p_^2^) are presented. Values are given as mean ± standard deviation for the different posturographic parameters depending on sensorimotor interventions (physiotherapy (PT), Galileo^®^, and Posturomed^®^). Variance analysis (group, time, interaction effects) for the two weeks of multimodal pain therapy. ALM: General linear model (*p*, η_p_^2^); effect size (d). F 1: Frequency band 1, visual and nigrostriatal system; F 2–4: Frequency band 2–4, peripheral vestibular system; F 5–6: Frequency band 5–6, somatosensory system; F 7–8: Frequency band 7–8, cerebellar system; WDI: weight distribution index; Synch.: Synchronization; Heel: forefoot-hindfoot ratio, percentage of weight distribution forefoot vs. hindfoot with a description of heel loading; Left: load on the left side, percentage of weight distribution left vs. right with the description of left side loading.

## Data Availability

Not applicable.

## Supplementary Materials

The following supporting information can be downloaded at https://www.mdpi.com/article/10.3390/jpm13040634/s1, Figure S1: Flow-Chart to the study. Figure S2: Galileo^®^. Figure S3: Posturomed^®^. Table S1: IBS parameters [30,31]. Table S2: Exercise program in physiotherapy. Table S3: Exercise program in Galileo^®^-Group. Table S4: Exercise program in Posturomed^®^-Group.
