# Supplementary material for: Effects of Different Forms of Sensorimotor Training on Postural Control and Functional Status in Patients with Chronic Low Back Pain
Source: J Pers Med. 2023 Apr 5;13(4):634. doi: 10.3390/jpm13040634 (PMC10145057; doi:10.3390/jpm13040634)
Supplement: Supplementary file 1 [file jpm-13-00634-s001.zip › jpm-2310426-supplementary.pdf]

Journal of *Personalized Medicine*

MDPI

Article

Effects of different forms of sensorimotor training on postural control and functional status in patients with chronic low back pain

Alex Rüger[1,2], Kevin Laudner[3], Karl-Stefan Delank[1], René Schwesig[1] and Anke Steinmetz[4*]

[1] Department of Orthopedic and Trauma Surgery, Martin-Luther-University Halle-Wittenberg, 06120 Halle, Germany; alex.rueger@uk-halle.de

[2] Department of Ophthalmology, Martin-Luther-University Halle-Wittenberg, 06120 Halle, Germany

[3] Department of Health Sciences, University of Colorado Colorado Springs, CO 80918, USA; klaudner@uccs.edu

[4] University Medicine Greifswald, Physical and Rehabilitation Medicine, Department of Trauma, Reconstructive Surgery and Rehabilitation Medicine, 17475 Greifswald, Germany; anke.steinmetz@med.uni-greifswald.de

*Correspondence: anke.steinmetz@med.uni-greifswald.de

Citation: Rüger, A.; Laudner, K.; Delank, K-S.; Schwesig, R.; Steinmetz, A. Effects of Different Forms of Sensorimotor Training on Postural Control and Functional Status in Patients with Chronic Low Back Pain. *J. Pers. Med.* **2023**, *13*, x. https://doi.org/10.3390/xxxxx

Academic Editor(s): Markus Gesslein, Eduard Kurz

Received: 13 March 2023
Revised: 28 March 2023
Accepted: 3 April 2023
Published: date

Copyright: © 2023 by the authors. Licensee MDPI, Basel, Switzerland. This article is an open access article distributed under the terms and conditions of the Creative Commons Attribution (CC BY) license (https://creativecommons.org/licenses/by/4.0/).

Abstract: The aim of this study was to compare three sensorimotor training forms in patients with chronic low back pain to determine their effects in the reduction of pain-related impairment and changes in posturography. Over two weeks, during multimodal pain therapy (MMPT) period, six sessions of either sensorimotor physiotherapy, training on the Galileo® or Posturomed® (n = 25 per group) were performed. A significant reduction in pain-related impairment after the intervention phase was shown across all groups (time effect: $p<0.001$; $\eta_p^2=0.415$). There was no change in postural stability (time effect: $p=0.666$; $\eta_p^2=0.003$), but there was a significant improvement in the peripheral vestibular system (time effect: $p=0.014$; $\eta_p^2=0.081$). An interaction effect was calculated for the forefoot-hindfoot ratio ($p=0.014$; 0.111). Only the Posturomed® group showed an improvement in anterior-posterior weight distribution (heel load: 47% vs. 49%). These findings suggest that these forms of sensorimotor training in the context of a multimodal pain therapy are suitable for reducing pain-related impairment. Posturography demonstrated stimulation of a subsystem, but no improvement in postural stability.

Keywords: Galileo®; Posturomed®; chronic, low back pain; sensory, motor, physiotherapy; rehabilitation

Sublementary Materials:

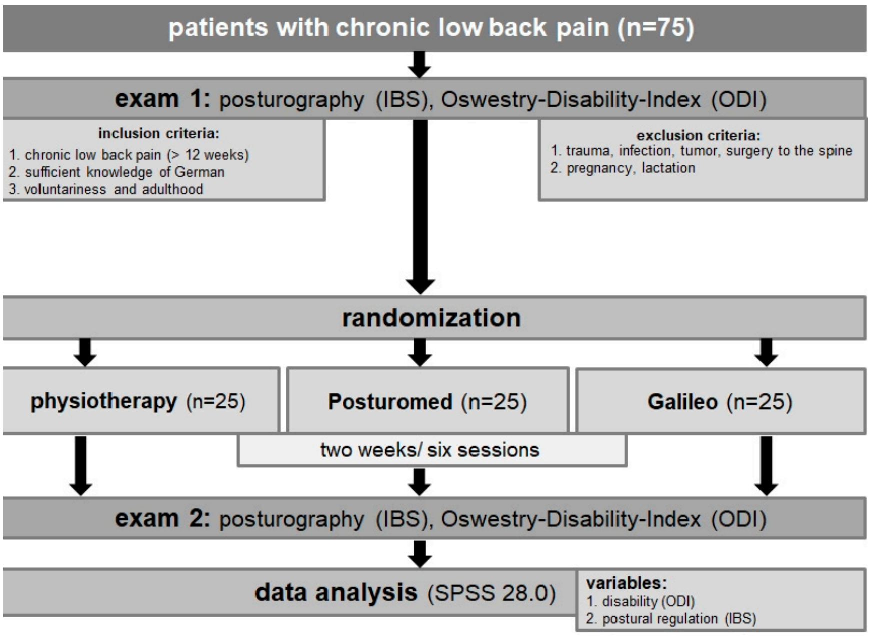

Figure S1: *Flow-Chart to the study*

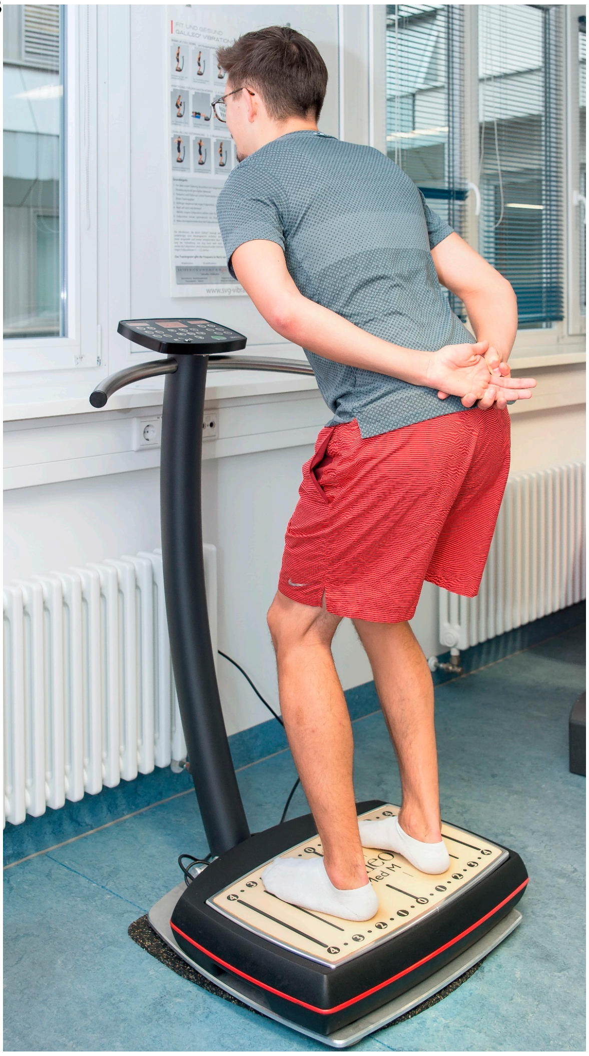

Figure S2: *Galileo®*

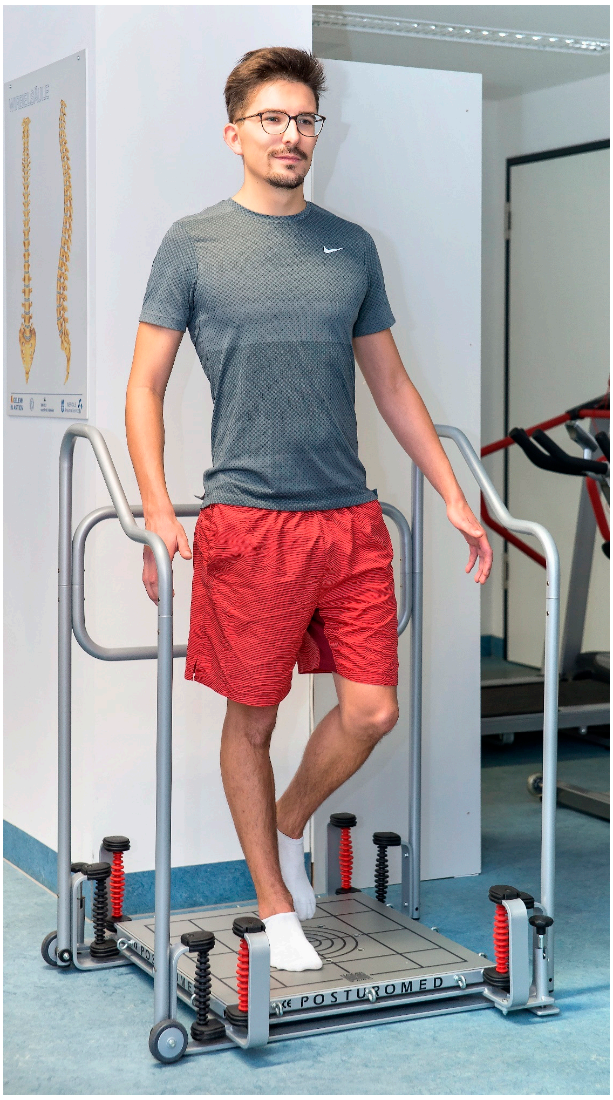

Figure S3: *Posturomed®*

Table S1: *IBS parameters [30,31]*

Category	Parameter	Interpretation/Explanation
Process parameters	Frequency band 1 (0.03–0.1 Hz)	Visual and nigrostriatal system
	Frequency band 2-4 (0.1–0.5 Hz)	Peripheral-vestibular system
	Frequency band 5-6 (0.5–1.0 Hz)	Somatosensory system
	Frequency band 7-8 (>1.0 Hz)	Cerebellar system
Product parameters	Stability indicator (ST)	The stability indicator (ST) was determined as the root mean square of the differences between

		pressure distributions on the plates and describes the postural stability. The larger the stability indicator, the higher the instability of the person is to be rated.
	Weight distribution index (WDI):	The WDI calculates the standard deviation in the weight distribution on the plates assuming that 25% of the body weight is distributed evenly across the four plates.
	Synchronization (Synch.)	Six values that describe the relationship of vibration patterns between plates calculated as a scalar product: 1000—complete coactivity; -1000—complete compensation; 0—no coactivity or compensation
	Forefoot–hindfoot ratio (Heel):	Percentage of load distribution between the forefoot and hindfoot with an emphasis on heel loading.
	Left–right ratio (Left)	Percentage of load distribution between the left and right feet with an emphasis on left side loading.

Table S2: *Exercise program in physiotherapy*

**Surface**	**Task**	**Duration/ Rest/Reps**
Stable	1. Stand upright on both feet, eyes open.	10 s/20 s/3
	2. Stand upright on both feet, eyes closed.	10 s/20 s/3
	3. Stand upright on one leg, eyes open.	10 s/20 s/3
	4. Stand upright on one leg, eyes closed.	10 s/20 s/3
	5. Stand upright on both feet, eyes open, pass ball around body at waist height (clockwise and counter-clockwise).	10 s/20 s/3
	6. Stand upright on both feet, eyes closed, pass the ball around the body at waist level (clockwise and counterclockwise).	10 s/20 s/3

	7. Stand upright on one leg, eyes open, pass the ball around the body at waist level (clockwise and counterclockwise).	10 s/20 s/3
	8. Stand upright on both feet, eyes open, throw the ball up (about 30cm) and catch it.	8 s/20 s/3
	9. Stand upright on one leg, eyes open, throw the ball up (about 30cm) and catch it.	8 s/20 s/3
Unstable (on a gymnastic mat)	10. Stand upright on both feet, eyes open.	10 s/20 s/3
	11. Stand upright on both feet, eyes closed.	10 s/20 s/3
	12. Stand upright on one leg, eyes open.	10 s/20 s/3
	13. Stand upright on one leg, eyes closed.	10 s/20 s/3
	14. Stand upright on both feet, eyes open, pass ball around body at waist height (clockwise and counterclockwise).	10 s/20 s/3
	15. Stand upright on one leg, eyes open, pass ball around body at waist height (clockwise and counterclockwise).	10 s/20 s/3
	16. Stand upright on both feet, eyes open, throw the ball up (about 30cm) and catch it.	8 s/20 s/3

Table S3*: Exercise program in Galileo®-Group*

Exercise Tasks	Position	Duration/Frequency/ Rest
1. Warm up and Acclimatization	Stand upright in the middle of the vibration plate, feet about shoulder-width apart, flexion in the hip/knee joint about 45°, extension of the ankle joint about 45° (basic position).	60 s/5 Hz/30 s
2. Muscle tension	Stand upright, move pelvis ventrally.	30 s/18 Hz/30 s
3. Muscle tension	Basic position.	30 s/15-25 Hz/30 s
4. Muscle tension	Lifting the heels.	30 s/15-25 Hz/60 s
5. Muscle tension	Arms crossed behind the head, pelvic movement in the plane from dorsal to ventral.	45 s/15-25 Hz/60 s
6. Back Coordination	Arms crossed behind the head, pelvic movement in the plane from dorsal to ventral.	60 s/5-7 Hz/60 s
7. Relaxation	Basic position, support via the bracket.	30 s/10 Hz/30 s

Table S4: *Exercise program in Posturomed®-Group*

Level	Locks	Exercise	Execution	Number of throws
1	Both closed	Step on the spot, one-legged stand.	Raise your feet alternately by approx. 10-15 cm in an upright position, hold every 3 changes in one-legged position for approx. 2 s.	0
2	Both closed	Throwing and catching in the medial sagittal plane.	Same as in level 1, with one-legged stand, but throw a ball (diameter 20cm, weight 100g) 1-5 times at a height of approx. 60-80 cm and catch it with both hands.	1-5
3	Both closed	Throwing and catching after rotation.	Same as in level 2, but rotate the leg during the one-legged stand 10-15° in the pelvic/shoulder line, then throw and catch the ball 2-6x ascending.	2-6
4	One opened	Step on the spot, one-legged stand.	Raise your feet alternately by approx. 10-15 cm in an upright position, hold every 3 changes in one-legged position for approx. 2 s.	0
5	One opened	Throwing and catching in the medial sagittal plane	Same as in level 4, but with the one-legged stand, throw a ball 1-5x at a height of approx. 60-80 cm and catch it with both hands.	1-5
6	One opened	Throwing and catching after rotation.	Same as in level 5, but rotate the leg during the one-legged stand 10-15° in the pelvic/shoulder line, then throw and catch the ball 2-6x ascending.	2-6
7	Both opened	Step on the spot, one-legged stand.	Raise your feet alternately by approx. 10-15 cm in an upright position, hold every 3 changes in one-legged position for approx. 2 s.	0
8	Both opened	Throwing and catching in the medial sagittal plane	Same as in level 7, but when standing on one leg throw a ball up 1-5x at a height of approx. 60-80 cm and catch it with both hands.	1-5

9	Both opened	Step on the spot, one-legged stand.	Same as in level 8, but rotate the leg during the one-legged stand 10-15°in the pelvis/shoulder line, then throw and catch the ball 2-6x ascending.	2-6
